# Association between metabolic score for visceral fat and obstructive sleep apnea: a cross-sectional study

**DOI:** 10.3389/fmed.2024.1480717

**Published:** 2024-12-12

**Authors:** Xue Xu, Jing Xu, Mengye Zhang

**Affiliations:** ^1^Department of Rehabilitation Medicine, The First Affiliated Hospital of Zhejiang Chinese Medical University (Zhejiang Provincial Hospital of Chinese Medicine), Hangzhou, Zhejiang, China; ^2^Department of Endocrinology, The Second Affiliated Hospital and Yuying Children's Hospital of Wenzhou Medical University, Wenzhou, China

**Keywords:** obesity, METS-VF, BMI, insulin resistance, weight, height

## Abstract

**Background:**

Previous studies have established a connection between obesity and obstructive sleep apnea (OSA), novel surrogate markers of adipose accumulation may serve as more critical and reliable factors for consideration. Consequently, this study aims to explore and elucidate the correlation between metabolic score for visceral fat (METS-VF) and OSA.

**Methods:**

In this cross-sectional study, the data from the National Health and Nutrition Examination Survey (NHANES) during the period from 2013 to 2020 were adopted. Through multivariate logistic regression, restricted cubic spline regression (RCS), subgroup analyses and sensitivity analyses, the correlation between METS-VF and OSA was explored.

**Results:**

Among 8,284 subjects, 4,176 of them were categorized as having OSA. It was observed that the quartile range of METS-VF increased, with a notable rise in the prevalence of OSA (32.8% vs. 49.8% vs. 56.9% vs. 62.1%, *p* < 0.001). Logistic regression analyses showed a significant positive correlation between METS-VF and the risk of having OSA, even after accounting for potential confounders (OR = 2.436, 95% CI: 2.065, 2.874). Subgroup analyses further revealed a stronger correlation between OSA and METS-VF among subjects who were female, younger, and Mexican Americans. RCS regression identified a positive linear correlation, without threshold effects. Sensitivity analyses with stop breathing (OR = 2.283, 95%CI: 1.169, 3.070) or snoring (OR = 2.716, 95%CI: 2.273, 3.246) as outcomes reaffirmed the positive correlation with METS-VF.

**Conclusion:**

Elevated METS-VF demonstrated a linear correlation with the increased incidence of OSA, suggesting the potential utility as a predictive index for OSA.

## Introduction

OSA is characterized by the complete or partial collapse of the upper airway for at least 10 s during sleep, leading to complete cessation (apnea) or decreased airflow (hypoventilation). Excessive daytime drowsiness is a prominent symptom of OSA (1, 2). Epidemiological data indicate that OSA affects approximately 17% of females and 34% of males aged 30 to 70 in the United States (3). Without any treatment in time, OSA will result in serious health complications such as cardiovascular diseases (4, 5), hypertension (6) and diabetes mellitus (DM) (7). Therefore, identifying precise and novel biomarkers for the early detection of OSA is crucial.

OSA is widely recognized as a significant complication correlated with obesity (8). Obesity is typified by the accumulation of visceral adipose tissues (VAT). Conventional metrics, such as body mass index (BMI), can only provide a general assessment on obesity and fail to distinguish visceral from subcutaneous fat. In recent developments, Bello-Chavolla et al. (9) introduced a novel visceral adiposity score, termed METS-VF, which is markedly superior to traditional obesity indexes in estimating VAT. METS-VF, encompassing variables such as WHtR, BMI, HDL-C, FPG, TG, gender, and age, can offer a comprehensive assessment on VAT and its metabolic implications, which can not only evaluate the glycolipid metabolism and distribution of body fat, but also incorporate gender and age-specific variations in VAT. Recent research has highlighted the superiority of METS-VF compared to traditional obesity indexes in predicting and assessing the risk of metabolic diseases, such as hyperuricemia, hypertension, DM, and chronic kidney dysfunction (CKD) (10–14). To date, however, no published studies have explored the correlation between METS-VF and the prevalence of OSA.

In this study, the data from the NHANES database were analyzed to explore the correlation between METS-VF and the prevalence of OSA in a nationally representative sample.

## Methods

### Research population

NHANES, conducted by NCHS (15), is a comprehensive study designed to assess the correlation between nutrition, health promotion, and disease prevention. The survey shall be conducted every 2 years by taking physical examinations, interviews, and various sections covering dietary, demographic, examination, and laboratory data.

**Figure 1:**
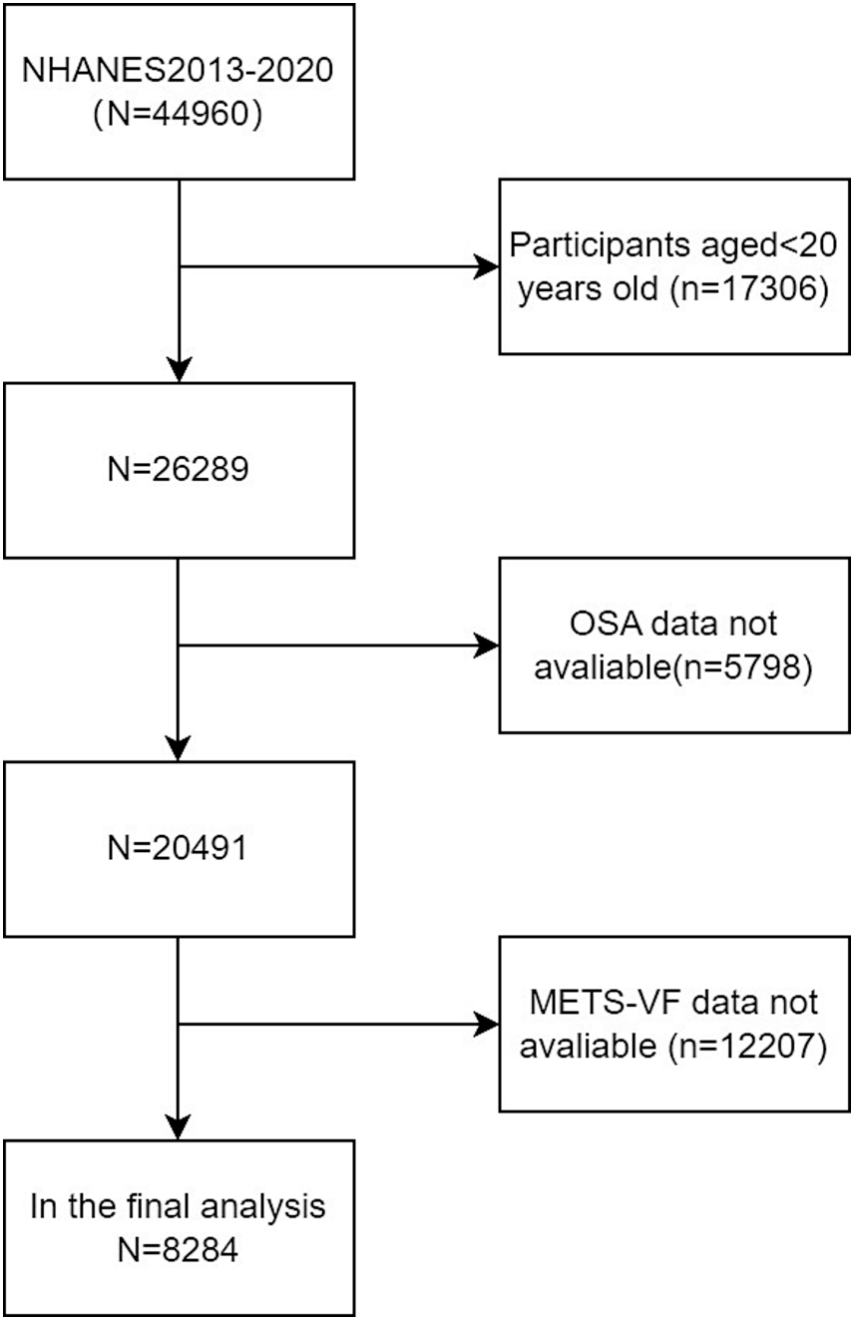
Flowchart of the sample selection from the 2013–2020 NHANES.

A total of 44,960 subjects were included in the NHANES database during the period from 2013 to 2020. By rigorous exclusion and inclusion criteria, 8,284 American adults from NHANES 2013–2020 were identified as samples. Specifically, 17,306 individuals under 20 years old, 5,798 individuals missing OSA data, and 12,207 individuals missing METS-VF were excluded from the study (as shown in Figure 1).

### Assessment of OSA

Consistent with prior research, high-risk for OSA was defined when an individual affirmed a positive response to at least one of the three questions of NHANES (16): (1) Daylight sleepy, characterized by excessive drowsiness while awake, despite sleeping for at least 7 h per night, reported between 16 to 30 times; (2) Stopped breathing, with episodes occurring three or more times per week; (3) Snoring: snoring on three or more occasions per week.

### Measurement of covariates

The demographics and lifestyle data came from the household interview questionnaires administered by highly trained medical personnel. Anthropometric indexes and biochemical parameters were obtained through medical examinations and subsequent laboratory assessments in the Mobile Examination Centre (MEC). According to previous studies (17, 18), potential confounding factors correlated with OSA and METS-VF were incorporated into the final analysis. These factors included demographic variables (age, height, race, blood pressure, gender, waist circumference, educational attainment, weight, and physical activities). Questionnaire surveys included alcohol consumption, hypertension, lipid-lowering drugs (LLDs) and DM. TC, UA, HbA1c, albumin, LDL-C, ALT, TG, GGT, AST, creatinine, and HDL-C were collected in blood samples. Less than 3% of values missed in total. Multiple imputation was performed for missing values. Self-reported race was categorized into the following five races: non-Hispanic Black, non-Hispanic White, other Hispanic, Mexican Americans, and other races. Educational level was divided into two levels: high school or above, less than high school. Alcohol consumption was assessed by using a question: “In 1 year, have you had at least 12 drinks of any type of alcoholic beverage?” Participants who answered ‘yes’ were identified as alcohol drinkers. Participants having diabetes mellitus were identified by having any of the following conditions: Have been told by a doctor or health professional having diabetes mellitus, HbA1c ≥ 6.5%, fasting plasma glucose≥7.0 mmoL/L, two-hour OGTT blood glucose≥11.1 mmoL/L, and use of diabetes mellitus medication or insulin. Hypertension in participants was defined based on any of the following: ever been told by a doctor or a health professional that had hypertension, mean systolic blood pressure ≥ 140 mmHg, and mean diastolic blood pressure ≥ 90 mmHg. Detailed measurements and data acquisition for each variable can be accessed at www.cdc.gov/nchs/nhanes.

### Calculation formula of METS-VF

The metabolic score for IR (METS-IR) was calculated with the following formula: METS-IR = Ln [TG (mg/dL) +2 × FPG (mg/dL)] × BMI (kg/m^2^) / Ln [HDL-C (mg/dL)] (19);

METS-VF was calculated with the following formula: METS-VF = 3.239 × [Ln (WHtR)]^3^ + 0.011 × [Ln (METS-IR)]^3^ + 0.319 × gender (male = 1, female = 0) +4.466 + 0.594 × [Ln (Age) (year)] (9).

### Statistical analysis

METS-VF values were categorized into quartiles (Q1: ≤6.27; Q2: 6.27–6.69; Q3: 6.69–7.00; Q4: ≥7.00). Categorical characteristics were expressed as proportions, whereas continuous variables were summarized by standard errors and means. Differences among quartile groups were assessed with chi-square tests or Kruskal-Wallis H test. Bonferroni test was adopted for the intergroup comparison. Variables demonstrating clinical and statistical significance in the univariate analyses (*p* < 0.05) were incorporated into the multivariate analyses. Multiple logistic regression models were employed to explore ORs and 95% CIs between OSA and METS-VF. The analysis incorporated three models: Model 1 (unadjusted), Model 2 (adjusted for age, race, and gender), and Model 3 (fully adjusted for drinking, educational level, TC, moderate physical activities, DM, albumin, SBP, DBP, ALT, AST, creatinine, GGT, LLDs and uric acid). Potential modifications of the correlation by covariates were explored with interaction tests and subgroup analyses. Additionally, whether the correlation between METS-VF and OSA was linear was determined through restricted cubic spline (RCS) analysis. Finally, the robustness of the findings were assessed through three sensitivity analyses: (1) Excluding subjects taking lipid-lowering drugs potentially affecting METS-VF, (2) taking “stopped breathing” as the dependent variable, and (3) taking “snoring” as the dependent variable. Data analyses were performed with R software (version 3.4.3) and Free Statistics software (version 1.9.2), with a significance threshold at *p* < 0.05 for all statistical tests.

## Results

### Baseline characteristics of subjects

**Figure 2:**
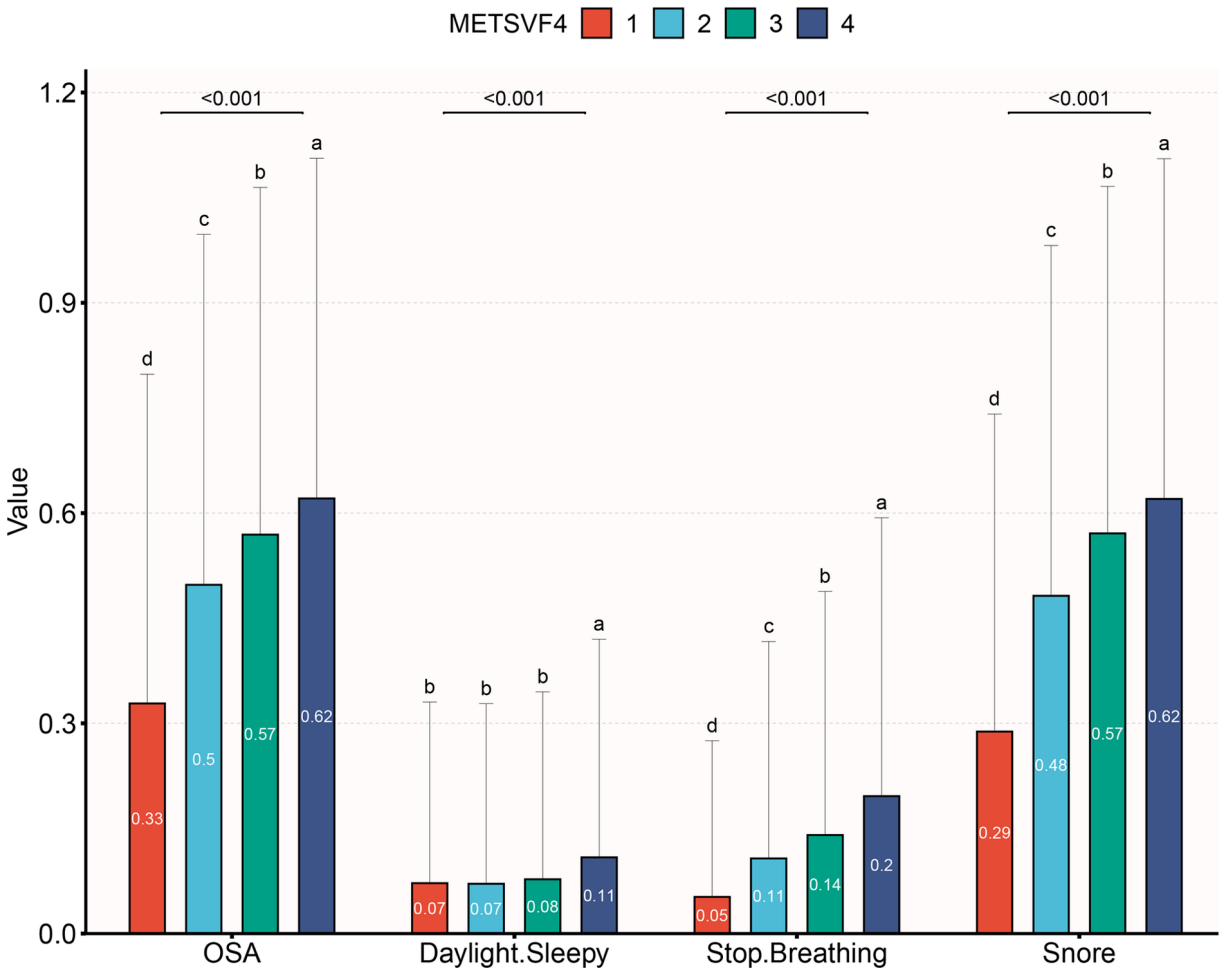
The prevalence of OSA and OSA symptom across quartiles of METS-VF.

A total of 8,284 subjects aged between 20 and 80 years old were included in this study, with a prevalence of OSA of 50.4%. Demographic characteristics, stratified by METS-VF quartiles, are presented in Table 1. Subjects in the highest METS-VF quartile exhibited a higher prevalence of DM, OSA, hypertension and elevated ALT, weight, uric acid, BMI, TG, SBP, waist circumference, and FPG, compared to those in the lowest quartile. Conversely, subjects in the highest quartile showed lower levels of HDL-C and albumin (*p* < 0.01) (as presented in Table 1). As illustrated in Figure 2, the prevalence of OSA increased across quartiles: 32.8% in Q1, 49.8% in Q2, 56.9% in Q3, and 62.1% in Q4, along with a rise in OSA symptoms such as daytime sleepiness, stopped breathing, and snoring.

**Table 1:** Weighted characteristics of the study population based on METS-VF quartiles.

Characteristic	Q1	Q2	Q3	Q4	*p* value
Number	2071	2068	2064	2081	
Age, year	37.0 ± 14.3	46.1 ± 15.7	54.0 ± 14.5	65.3 ± 10.6	<0.001
Race, *n*%					<0.001
Mexican American	198 (9.6)	337 (16.3)	339 (16.4)	299 (14.4)	
Other Hispanic	165 (8.0)	236 (11.4)	258 (12.5)	256 (12.3)	
Non-Hispanic White	636 (30.7)	658 (31.8)	666 (32.3)	852 (40.9)	
Non-Hispanic Black	518 (25.0)	420 (20.3)	488 (23.6)	498 (23.9)	
Other Race	554 (26.8)	417 (20.2)	313 (15.2)	176 (8.5)	
Moderate activities, *n*%					<0.001
Yes	1,008 (48.7)	909 (44)	862 (41.8)	632 (30.4)	
No	1,063 (51.3)	1,159 (56)	1,201 (58.2)	1,447 (69.6)	
Diabetes, *n*%					<0.001
Yes	47 (2.3)	178 (8.8)	367 (18.4)	718 (36.2)	
No	1990 (97.7)	1839 (91.2)	1,627 (81.6)	1,267 (63.8)	
Hypertension, *n*%					
Yes	279 (13.5)	602 (29.2)	886 (43)	1,365 (65.6)	
No	1789 (86.5)	1,462 (70.8)	1,174 (57)	716 (34.4)	
Education level, *n*%					<0.001
Less than high school	271 (13.1)	411 (19.9)	480 (23.3)	522 (25.1)	
High school or above	1800 (86.9)	1,657 (80.1)	1,584 (76.7)	1,559 (74.9)	
Drinking, *n*%					0.502
Current or ever, %	1,279 (61.8)	1,314 (63.5)	1,278 (61.9)	1,319 (63.4)	
Never	792 (38.2)	754 (36.5)	786 (38.1)	762 (36.6)	
LLDs, %	78 (3.8)	302 (14.6)	512 (24.8)	911 (43.8)	<0.001
Male, *n*%	1,079 (52.1)	1,094 (52.9)	974 (47.2)	868 (41.7)	<0.001
OSA, *n*%	680 (32.8)	1,029 (49.8)	1,175 (56.9)	1,292 (62.1)	<0.001
Weight, cm	66.7 ± 13.5	80.0 ± 17.3	87.7 ± 21.8	96.4 ± 22.4	<0.001
Body mass index, Kg/m^2^	23.2 ± 3.3	28.3 ± 4.7	31.6 ± 6.3	35.6 ± 6.9	<0.001
Height, cm	169.0 ± 9.7	167.5 ± 9.7	166.0 ± 10.1	164.2 ± 9.8	<0.001
Waist circumference, cm	82.5 ± 8.5	97.0 ± 9.8	106.0 ± 12.9	116.8 ± 13.3	<0.001
Systolic blood pressure, mmHg	116.1 ± 14.9	123.7 ± 18.3	128.8 ± 18.8	134.6 ± 19.6	<0.001
Diastolic blood pressure, mmHg	68.6 ± 11.2	71.5 ± 12.3	72.7 ± 13.1	69.3 ± 14.8	<0.001
FPG, mmol/L	5.49 ± 1.11	6.05 ± 1.95	6.48 ± 2.18	7.18 ± 2.62	<0.001
ALT, U/L	20.2 ± 20.1	24.4 ± 17.8	24.9 ± 17.0	22.6 ± 14.9	<0.001
AST, U/L	22.7 ± 18.4	23.0 ± 13.2	23.1 ± 11.8	22.7 ± 20.8	0.832
GGT, U/L	24.1 ± 34.2	32.0 ± 41.9	34.1 ± 65.2	35.0 ± 44.9	<0.001
Albumin, g/dl	4.24 ± 0.35	4.11 ± 0.36	4.05 ± 0.33	3.96 ± 0.34	<0.001
Creatinine, umol/L	74.0 (61.0, 86.0)	72.0 (60.0, 86.0)	72.0 (61.0, 86.0)	77.0 (64.0, 94.0)	<0.001
Uric acid, umol/L	291.5 (243.9, 345.0)	315.2 (261.7, 368.8)	321.2 (267.7, 386.6)	345.0 (291.5, 404.5)	<0.001
Total cholesterol, mmol/L	4.69 ± 1.01	5.02 ± 1.08	4.99 ± 1.14	4.74 ± 1.11	<0.001
Triglycerides, mmol/L	0.87 (0.66, 1.22)	1.22 (0.82, 1.77)	1.29 (0.95, 1.87)	1.42 (1.06, 1.90)	<0.001
HDL-cholesterol, mmol/L	1.56 ± 0.46	1.38 ± 0.43	1.34 ± 0.41	1.32 ± 0.36	<0.001
LDL-cholesterol, mmol/L	2.68 ± 0.85	3.01 ± 0.91	2.98 ± 0.96	2.75 ± 0.95	<0.001
METS-VF	5.66 ± 0.52	6.50 ± 0.12	6.85 ± 0.09	7.16 ± 0.10	<0.001

Values are mean ± SD or number (%). *p* < 0.05 was deemed significant. BMI, body mass index; FPG, fasting blood glucose; TC, total cholesterol; TG, triglyceride; HDL-c, High density lipoprotein cholesterol; LDL-c, Low density lipoprotein cholesterol; LLDs, lipid-lowering drugs; GGT, glutamyl transpeptidase; METS-VF, metabolic score for visceral fat.

### Correlation between METS-VF and metabolic parameters

Spearman’s correlation analysis (as presented in Table 2) revealed that METS-VF was positively correlated with FPG, DBP, TG, uric acid, SBP, uric acid and LDL-C, and negatively correlated with HDL-C (all *p* < 0.05).

**Table 2:** Spearman’s correlation of METS-VF levels with clinical and biochemical parameters.

Variable	METS-VF	
	*r*	*p*
SBP	0.393	<0.001
DBP	0.046	0.003
FPG	0.454	<0.001
TC	0.018	0.104
TG	0.339	<0.001
HDL-C	−0.212	<0.001
LDL-C	0.027	0.015
Uric acid	0.238	<0.001

### Logistical correlation between METS-VF and OSA

**Figure 3:**
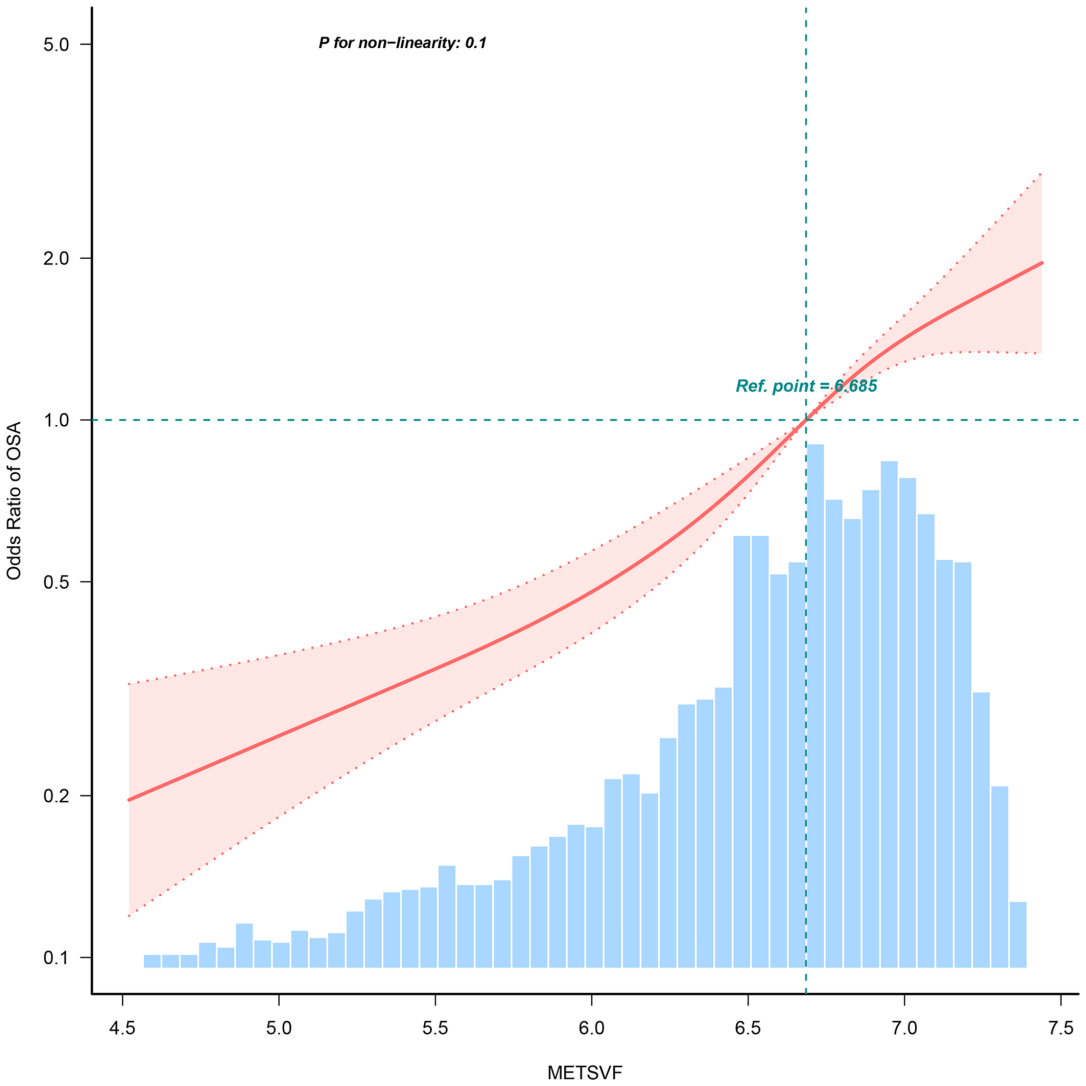
Restricted cubic spline fitting for the association between METS-VF index levels and OSA.

In order to explore the correlation between OSA and METS-VF, three multiple regression models were developed (as presented in Table 3). Model 1, the unadjusted model, indicated a statistically significant positive correlation between OSA and METS-VF, which remained evident after adjusting for all covariates in Model 3 (OR = 2.436, 95% CI: 2.065, 2.874, *p* < 0.001). According to the sensitivity analysis, METS-VF was categorized into quartiles, showing that in the fully adjusted Model 3, subjects in the second, third, and fourth quartiles had a statistically significant increase in the risk of having OSA by 0.945, 1.601, and 2.481, respectively, compared to those in the lowest quartile. To further explore the correlation between METS-VF and OSA, restricted cubic spline smoothing curve fitting with Model 3 was conducted. The results depicted in Figure 3 revealed a linear correlation between METS-VF and OSA, without threshold effects.

**Table 3:** Association between METS-VF and OSA in logistic regression analysis.

	Model1 OR (95% CI) *p* value	Model II OR (95% CI) *p* value	Model III OR (95% CI) *p* value
METS-VF	2.188 (2.024, 2.365), <0.001	2.740 (2.478, 3.030), <0.001	2.436 (2.065, 2.874), <0.001
METS-VF (Quartile)			
Q1	Reference	Reference	Reference
Q2	2.026 (1.786, 2.298), *p* < 0.001	2.206 (1.934, 2.517), <0.001	1.954 (1.607, 2.376), <0.001
Q3	2.704 (2.382, 3.068), *p* < 0.001	3.246 (2.815, 3.743), <0.001	2.601 (2.087, 3.243), <0.001
Q4	3.350 (2.949, 3.805), *p* < 0.001	4.591 (3.897, 5.408), *p* < 0.001	3.481 (2.678, 4.526), <0.001
P for trend	<0.001	<0.001	<0.001

Model I: None covariates were adjusted; Model II: gender, age and race were adjusted; Model III: gender, age, race, drinking, educational level, TC, moderate physical activities, diabetes, albumin, SBP, DBP, ALT, AST, creatinine, GGT, LLDs and uric acid were adjusted.

### Subgroup analysis

**Figure 4:**
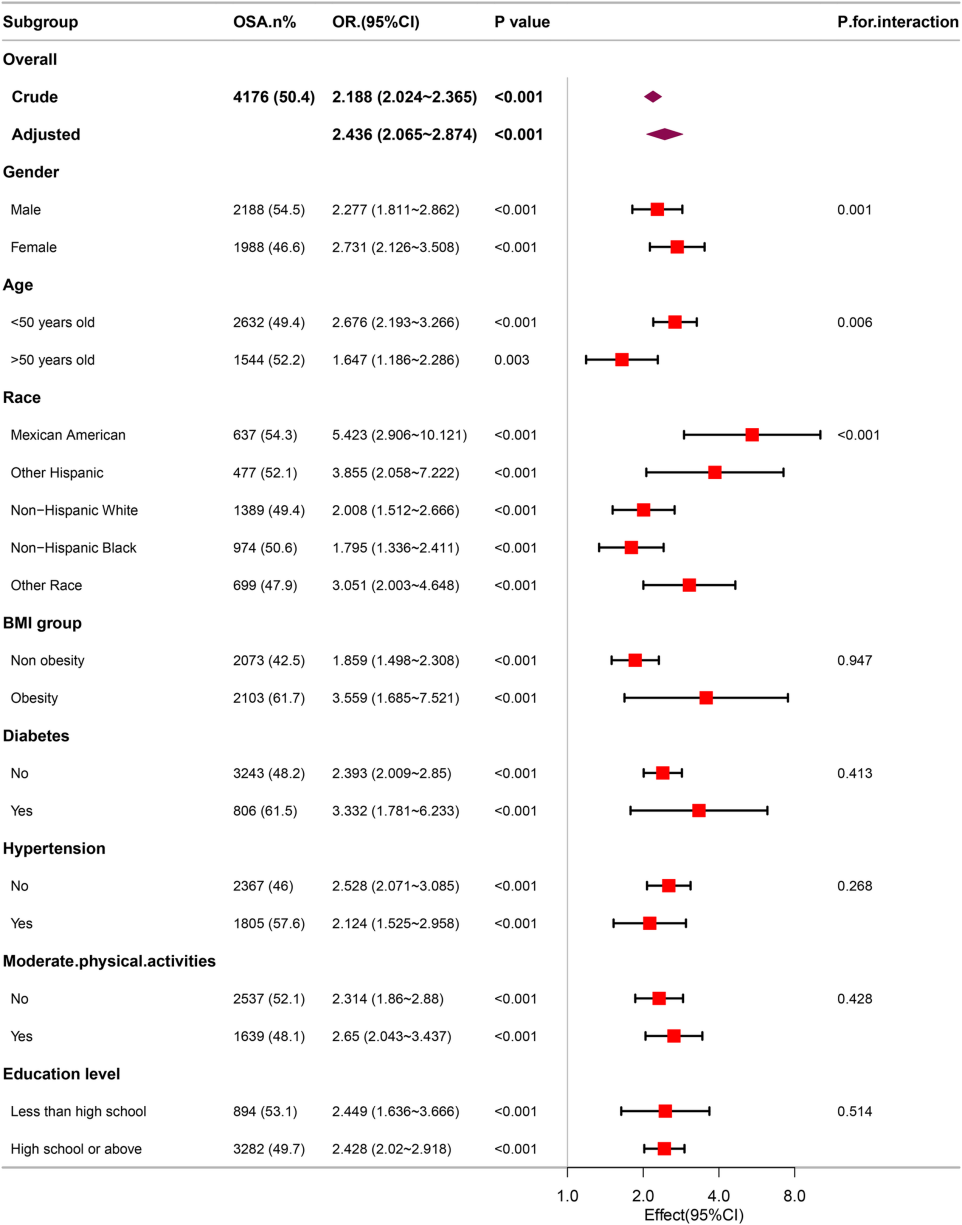
Association between METS-VF and the risk of OSA in various subgroups.

Through comprehensive subgroup analyses and interaction tests, the robustness of the correlation between METS-VF and OSA was evaluated, to identify potential population variations (as shown in Figure 4). The results consistently demonstrated a notable correlation between METS-VF and OSA within various subgroups. It is particularly noteworthy that there were significant interaction effects between METS-VF and age, gender and race (all interaction *p* < 0.05). The correlation between METS-VF and OSA was more pronounced in subjects who were female, younger, and Mexican Americans.

### Sensitivity analyses

The results of the sensitivity analysis are presented in Table 4. Sleep-related outcomes were taken as dependent variables in the adjusted Model 3, finding that METS-VF was correlated to stopped breathing (OR, 2.283; 95% CI, 1.697, 3.070) and snoring (OR, 2.716; 95% CI, 2.273, 3.246). After excluding subjects who received lipid-lowering drugs, the correlation between METS-VF and OSA remained stable (OR, 2.493; 95% CI, 2.091, 2.974).

**Table 4:** Sensitivity analyses.

	OSA or OSA symptom, *n* (%)	Adjusted OR (95 CI%)
Sensitivity analyses^1^		
METS-IR (continuous)	963 (11.6)	2.283 (1.697, 3.070), <0.001
METS-IR		
Q1	105 (5.1)	1.00 (Ref)
Q2	211 (10.2)	2.141 (1.493, 3.069), <0.001
Q3	273 (13.2)	2.721 (1.846, 4.012), <0.001
Q4	374 (18)	4.043 (2.597, 6.293), <0.001
Sensitivity analyses^2^		
METS-IR (continuous)	3,782 (45.7)	2.716 (2.273, 3.246), <0.001
METS-IR		
Q1	565 (27.3)	1 (Ref)
Q2	934 (45.2)	2.147 (1.748, 2.637), <0.001
Q3	1,093 (53)	2.921 (2.316, 3.683), <0.001
Q4	1,190 (57.2)	3.834 (2.910, 5.051), <0.001
Sensitivity analyses^3^		
METS-IR (continuous)^3^	3,097 (47.8)	2.493 (2.091, 2.974), <0.001
METS-IR		
Q1	637 (31.9)	1 (Ref)
Q2	856 (48.4)	1.941 (1.583, 2.380), <0.001
Q3	874 (56.3)	2.669 (2.109, 3.378), <0.001
Q4	730 (62.6)	3.852 (2.867, 5.175), <0.001

^1Stopped breathing was used as the dependent variable. 2Snoring was utilized as the dependent variable. 3Participants taking Lipid-lowering drugs that potentially affect METS-VF were excluded.^

## Discussion

This cross-sectional study encompassing 8,284 representative adults identified a notable positive correlation between METS-VF and the probability of having OSA. This correlation was particularly pronounced among subjects who were female, younger and Mexican Americans. Notably, linear correlation was identified between OSA and METS-VF, without threshold effects.

METS-VF, a novel estimator of VAT recently developed by Bello Chavolla OY et al. (9) has been meticulously developed and validated, as documented in prior literature. Due to the computational simplicity and high accuracy of METS-VF in predicting visceral obesity, increasing researchers have explored and validated its superior efficacy in assessing and predicting the risk of having the diseases correlated with visceral obesity. In the study, Yu et al. demonstrated that METS-VF can exhibit a robust predictive capacity for CKD compared to other markers of central adiposity (10). Additionally, compared to other obesity evaluation indexes, METS-VF can exhibit both applicability and reliability as a predictor of DM and hypertension within Chinese population (11, 12). A study involving 36,876 subjects identified a positive correlation between asthma and METS-VF (20). For non-obese females, METS-VF has been proven to be beneficial in guiding the management and prevention of hyperuricemia (13). Numerous studies have corroborated the strong correlation between these diseases and OSA (21–23). These findings provide indirect evidence of the robust diagnostic capability of METS-VF for identifying OSA. This study revealed a significant linear positive correlation between METS-VF and the probability of having OSA in a nationally representative sample for the first time.

The correlation between OSA and obesity is characterized by a complex interdependence (8). Notably, obesity, particularly the accumulation of excess abdominal fat, is a major risk factor for the exacerbation and development of OSA. Abdominal obesity can not only elevate intra-abdominal pressure and reduce lung volume, thereby heightening the risk of upper airway collapse (24), but it is also correlated with an increase in visceral fat, secreting various inflammatory and adipose-derived factors, leading to oxidative stress and systemic inflammation. These processes affect muscle activities in the upper respiratory tract, promote the proliferation of adipose tissues around the upper respiratory tract, and thus increase the risk of having OSA (25, 26). In addition, irregular sleep patterns and frequent awakenings correlated with OSA can disrupt hormonal regulation, leading to increased hunger and a preference for high-calorie foods (27–29). Additionally, evidence indicates that OSA can alter the lipid profile (30), which can exacerbate lipid abnormalities by enhancing inflammatory responses. And OSA itself can exacerbate lipid abnormalities by increasing IR and inflammatory responses, thereby creating a negative feedback loop (31, 32). Furthermore, aging is correlated with the accumulation of senescent adipocytes in adipose tissues, leading to disruptions in lipid metabolism, glucose metabolism, immune regulation and endocrine function, within adipose tissues (33, 34). METS-VF can incorporate the aforementioned parameters (WHtR, age, lipid profile, and insulin resistance) to evaluate the metabolic status of visceral fat, which may can partially indicate the risk of having OSA.

Subgroup analyses in this study have uncovered a novel finding that elevated METS-VF was significantly correlated with an increased prevalence of OSA in individuals under 50 years old. This increased risk may be correlated with age-related physiological and metabolic changes, including alterations in lipid distribution and related metabolic markers (35, 36). Older adults are more prone to have hypertension, cardiovascular diseases, and DM, which can affect both metabolic indexes and sleep quality, which may consequently attenuate the correlation between METS-VF and OSA. Notably, Hai Deng et al. (8) have elucidated this phenomenon by proposing that the divergent effects of adipose tissue distribution in older adults may account for this discrepancy. Furthermore, it was identified that gender influences the correlation between METS-VF and OSA. Females typically exhibit a higher proportion of body fat compared to males and experience a reduction in estrogen levels after menopause, which may increase the risk of having OSA during menopause (37, 38).

This study carries important implications for clinical practice, particularly given the increasing annual prevalence of cardiovascular and cerebrovascular diseases correlated with OSA (39). OSA has emerged as a notable health issue impacting public well-being. Nevertheless, the diagnosis of OSA is frequently a lengthy, resource-intensive, and costly process for patients. Therefore, there is a pressing clinical necessity to pinpoint a convenient and effective diagnostic approach for OSA. METS-VF, a cost-efficient and readily measurable metric, satisfactorily fulfills these clinical needs. The results of this study provide important insights for healthcare professionals in efficiently evaluating the risk of having OSA in patients.

The study’s primary strength is its distinction as the first cross-sectional analysis to explore the correlation between METS-VF and OSA, supported by a sufficiently large and representative sample size. However, it is crucial to acknowledge the limitations inherent in this study. Firstly, the establishment of a causal relationship between METS-VF and OSA was not feasible through cross-sectional studies. As discussed above, a bidirectional relationship may exist. Secondly, its reliance on data solely from US adults, which may impede the generalizability of the results to other populations. Thirdly, there are numerous potential influencing factors for OSA and METS-VF. Although as many relevant covariates as possible have been incorporated into the models, it is still challenging to completely exclude the influence of other potential covariates, such as diet and genetic factors. Fourthly, in this study, the risk of having OSA was assessed through three questions, which suggests a high risk of having OSA rather than a confirmed diagnosis, and also lead to recall bias. Ideally, diagnosing OSA requires overnight polysomnography or polygraphy. Future research should incorporate prospective cohort studies and richer datasets to overcome these limitations and should also aim at uncovering the underlying mechanisms linking these conditions.

## Conclusion

This study revealed a notable correlation between elevated METS-VF and OSA. METS-VF can function as an independent predictor of OSA, aiding in early detection and diagnosis to mitigate the risks correlated with the conditions.

## Data Availability

Publicly available datasets were analyzed in this study. This data can be found here: NHANES, http://www.cdc.gov/nhanes.
